# Gramado Declaration: The Impact of 20 Years of Cardiovascular Prevention

**DOI:** 10.5935/abc.20170040

**Published:** 2017-03

**Authors:** Aloyzio Achutti, Ricardo Stein, Lúcia Pellanda, Bruce B. Duncan

**Affiliations:** 1 Universidade Federal do Rio Grande do Sul (UFRGS), Porto Alegre, RS - Brazil.; 2 Universidade Federal de Ciências da Saúde de Porto Alegre (UFSCPA), Porto Alegre, RS - Brazil.

**Keywords:** Cardiovascular Diseases / prevention & control, Cardiovascular Diseases / epidemiology, Cardiovascular Diseases / trends

From May 1st to 10th, 1997, the First Brazilian Seminar on Cardiovascular Epidemiology,^1^ was held in the city of Gramado (RS), in the manner in which the World Federation of Cardiology has been promoting in various parts of the world since 1968 with The title Ten-Day International Didactic Seminars on Cardiovascular Epidemiology and Prevention.^2^

The initiative came from the Scientific Advisory Board of the Faculty of Medicine of the Universidade Federal of Rio Grande do Sul (UFRGS) and the Cardiology Clinical Department and of the Committe de Epidemiology and Public Health of the *Sociedade Brasileira de Cardiologia* (SBC), under the auspices of the Coordination of Higher Education for Personal Development (CAPES), the World Heart Federation (at that time still called the *International Society and Federation of Cardiology*) and the Inter-American Heart Foundation.

Together with the two coordinators, Aloyzio Achutti and Bruce Duncan, several national teachers (Annick Fontbonne, Eduardo de Azeredo Costa, Emilio Moriguchi, Jorge Pinto Ribeiro, Maria Inês Reinert Azambuja, Maria Inês Schmidt, Paulo Lotufo, Rosely Sichieri and Sérgio Bassanesi), and three international guests (Teri Manolio, Director of Epidemiology and Biometrics of the National Heart Lung and Blood Institute, Ulrich Grueninger, Head of Research and Medical Education of the Swiss Federal Office of Public Health, and Woody Chambless of the Department of Biostatistics at the University of North Carolina) ministered the activities. The 40 participants were from 10 Brazilian states.

In addition to basic concepts of epidemiology and statistics, and topics related to etiology and the prevention of cardiovascular diseases, were part of the program issues that, although currently consecrated, were new in Brazil at the time, as medicine based in evidence and systematic/meta-analysis review. At the time of the beginning of the implantation of the Unified Health System (SUS) and the concern with chronic noncommunicable diseases as a public health problem, this unique meeting enabled and encouraged Brazilian leaders in the field of cardiovascular prevention - several of whom later assumed positions of national leadership. There was a wide debate and, from the first day, time was made to the elaboration of a document that presented three different perspectives of prevention: individual, local and demographic. This document was called the Gramado Declaration^3^ and was widely nationally and internationally disseminated.

For the consolidation of the document, an online discussion was conducted through e-mail - which, at that time, was used by only 23 participants. From this experience, with messages that began with the "dear friends of the heart" greeting, a social group was started that was named AMICOR, at the suggestion of Eduardo de Azeredo Costa.^4^ In the course of time a website was created, and the AMICOR designation was also used by ProCOR, released two months later, during the Third International Conference on Preventive Cardiology, on the initiative of Professor Bernard Lown (Boston, USA). The name AMICOR was also adopted for some time by SBC on its website, under the name ProCOR / AMICOR, and later in 2004, as a blog named AMICOR.

Since then, much has happened, in terms of Brazilian public health. However, ischemic heart disease remains the main cause of morbidity and mortality in Brazil^5^, and social inequalities continue to have direct and indirect impact on early mortality due to cardiovascular diseases in our country.^6-8^ In the beginning of 2017, when the Declaration Of Gramado completes 20 years, some evils that affect Brazilian public health show that there is much to be done in the short, medium and long term to face with major achievement the overwhelming burden of cardiovascular disease in Brazil.

On the other hand, as it was already evident in the Seminar and it is increasingly clear nowadays, that cardiovascular diseases can be prevented by public health actions that involve the control of risk factors, as well as by the optimized clinical management of patients. When checking the website of the Global Burden of Disease, it is observed that the mortality standardized by cardiovascular diseases in Brazil from 1995 to 2015 fell by 36%.^9^ Recent calculations using slightly different methodology suggest even greater decline - over 2% per year.^10^ This reduction can be observed in different Brazilian studies, in various contexts and age groups.^11-15^

It is always difficult to assign causes for changes in disease incidence at the population level. However, improvements such as those that have been seen are, in part, the result of thousands of small gains from multiple actions and actors in the health sector. We would like to consider that the Gramado Seminar, held in the distant year of 1997, was one of these actions and may have contributed to the advances of practical impact seen in the cardiovascular health of the population.

The reduction of cardiovascular diseases in Brazil and in the world is a complex task that depends on numerous agents and continuous effort. Thus, in 2012, was published in the *Brazilian Cardiology Archives* the "Carta do Rio de Janeiro",^16^ prepared under the auspices of SBC during the III Brazil Prevent / I Latin America Prevent , endorsing the overall target of 25% reduction in early mortality from noncommunicable diseases up to 2025, set out in the World Health Assembly (WHA). The letter was signed by SBC, the *Sociedad Interamericana de Cardiologia*, the American Heart Association, the European Society of Cardiology and the World Heart Federation, and has made concrete decisions on how to achieve global goals.

Among these deliberations, many could already be observed as fundamental since the Gramado Declaration, such as "Implementing actions to acquire epidemiological information, including mortality and cardiovascular morbidity, execution and maintenance of existing registries in some of the signatories, aiming at development of strategies that promote the planning of health actions" and "Create an international permanent discussion forum to monitor the actions aimed at prevention, diagnosis and treatment of Cardiovascular risk factors in Latin America ", of which the AMICOR group could be considered an embryo.

As stated at the end of the Gramado Declaration.^3^ "Finally, despite the enormous scientific and technological advances already achieved or prospective in cardiology, it is increasingly necessary to construct a paradigm of health and disease that allows the benefit of such achievements to the entire population. Therefore, a reform in medical education and education of other health professionals is necessary, along with a broad discussion in which popular culture participates, contributing to the evolution of the assistance model, from the traditional biomedical to the biopsychosocial, with emphasis In health and not only in disease".

Thus, it is up to all of us to maintain the mobilization for effective and evidence-based cardiovascular prevention, taking into account the values of society. Actions such as the Brazilian Seminar, with in-depth discussion of relevant topics and strategic objectives, can multiply and have a significant impact in the long run.
